# Dietary supplementation with *Bacillus subtilis PB6* alleviates diarrhea and improves growth performance and immune function in weaned piglets fed a high-protein diet

**DOI:** 10.3389/fvets.2025.1525354

**Published:** 2025-02-18

**Authors:** Yang Liu, Lei Cao, Chenhao Yu, Qiang Zhou, Hua Li, Ruinan Zhang, Jiayong Tang, Zhiming Zhang, Zheng Luo, Xuemei Jiang, Zhengfeng Fang, Yan Lin, Shengyu Xu, Yong Zhuo, Lun Hua, De Wu, Bin Feng, Lianqiang Che

**Affiliations:** ^1^Key Laboratory for Animal Disease-Resistant Nutrition of the Ministry of Education of China, Animal Nutrition Institute, Sichuan Agricultural University, Chengdu, China; ^2^Kemin (China) Technologies Co., Ltd., Zhuhai, China

**Keywords:** probiotics, nutrient digestibility, inflammation, weaning stress, gut microbiota

## Abstract

This study aimed to evaluate the effects of dietary supplementation with *Bacillus subtilis PB6* on growth performance, diarrhea scores, nutrient digestibility, immune function, and gut microbiota in weaned piglets fed a high-protein diet. A total of 96 weaned piglets were randomly divided into three groups in a randomized complete block design and received a low-protein diet (LP, 18.27% crude protein), a high-protein diet (HP, 20.97% crude protein), or a high-protein diet supplemented with probiotics (HPPRO, *B. subtilis PB6* spores 2 × 10^11^ CFU/ kg) for 21 days. Each group had eight replicates with four piglets per replicate. The results showed that piglets fed the HPPRO diet had significantly higher average daily gain and average daily feed intake during days 8–14, days 15–21, and throughout the experimental period than piglets fed the LP diet (*p* < 0.01). In parallel, piglets fed the HPPRO diet had lower feed-to-gain ratio (F:G) values during days 8–14 than piglets fed the LP diet (*p* < 0.05). Piglets fed the HP diet had increased diarrhea scores than piglets fed the LP diet (*p* < 0.01), but the diarrhea scores of piglets fed the HPPRO diet were lower than those of the HP piglets (*p* < 0.01), which had the lowest nutrient digestibility. Moreover, piglets fed the HPPRO diet had lower plasma concentrations of haptoglobin than HP piglets (*p* < 0.05) and lower pig major acute-phase protein levels than HP and LP piglets (*p* < 0.05). The downregulation of genes (toll-like receptor-4, tumor necrosis factor-*α* [TNF-α], and TNF receptor-associated factor-6) in the ileal tissue associated with inflammation was observed in HPPRO-fed piglets compared to LP- and HP-fed piglets (*p* < 0.05). Piglets fed the HPPRO diet had increased relative abundance of genera related to proteolysis, such as g_*Fusobacterium* and g_*Acidaminococcus*, and genera related to butyrate production, such as g_*Anaerostipes* and g_*Megasphaera*. Furthermore, piglets fed the HPPRO diet had a higher concentration of butyrate in the colonic digesta than piglets fed the LP diet (*p* < 0.05). In conclusion, piglets fed the high-protein diet supplemented with 300-mg/kg *B. subtilis PB6* had better growth performance, which was associated with relatively higher nutrient digestibility, an improved intestinal bacterial profile, and a lower inflammatory response.

## 1 Introduction

After weaning, the nutrient source of piglets shifts from highly digestible liquid milk to a solid dry diet, which often leads to diarrhea due to undigested proteins, antinutritional factors in the feed (1), and the immature digestive system of piglets (2). In the past two decades, it has become common practice to reduce the dietary protein level of the feed while supplementing essential amino acids to alleviate digestive dysfunction. Currently, in commercial feed, the crude protein (CP) level for weaned piglets is typically below 20%. However, it should be noted that the primary nutrient source for suckling piglets, the milk protein level of sows, is more than 25% dry matter (DM) (3), indicating that weaned piglets are capable of digesting more protein if it is of high protein quality. In contrast, the lower amount of intact protein in the low-protein diet supplemented with crystalline amino acids may be insufficient to maximize the growth potential of piglets (4).

For weaning piglets, however, the practical conflict between high intact protein requirements and diarrhea induced by high dietary protein levels cannot be ignored and needs to be resolved. It should be noted that the undigested and unabsorbed proteins reach the hindgut and are fermented by the distal intestinal flora to generate toxic nitrogen metabolites, such as ammonia, amines, hydrogen sulfide, and *N*-nitroso compounds (5), increasing the risk of postweaning diarrhea in piglets. In parallel, gut microbiota dysbiosis, induced by abrupt dietary transition and environmental changes at weaning, is also recognized as one of the keys leading to the etiology of postweaning diarrhea and enteric infections (6). Protein fermentation in the hindgut may also be associated with an increased abundance of pathogenic bacteria (7).

As a Gram-positive bacterium that produces spores and displays resistance to various environmental stresses, *Bacillus subtilis* preserves excellent viability and stability during gastrointestinal transit (8). In addition, *B. subtilis* has the potential to release exoenzymes to digest dietary protein (8). Meanwhile, the metabolites of *B. subtilis* stimulate the biosynthesis of protease in the pancreas or hepatopancreas, inducing the secretion of endogenous digestive enzymes in the gastrointestinal tract and activating protease activities and pancreatic trypsin (9). It has been suggested that B. subtilis increased the expressions of intestinal tight junction proteins, indicating the role in maintaining intestinal barrier function (10). In this study, therefore, we aimed to evaluate the effects of a high-protein diet supplemented with *B. subtilis PB6* on growth performance, diarrhea scores, nutrient digestibility, immune function, and gut microbiota in weaned piglets.

## 2 Materials and methods

### 2.1 Ethical approval

The experiment was performed following the animal protection law (ethic approval code: SICAU 2023314113) and was performed in accordance with the Guide for Animal Care and Use approved by the Sichuan Agricultural University Institutional Animal Care and Use Committee.

### 2.2 Animals and experimental design

A total of 96 piglets (Duroc × Landrace × Yorkshire), weaned at 21 ± 2 days of age with an initial body weight (BW) of 6.12 ± 0.30 kg, were randomly assigned to 3 dietary treatments in a randomized complete block design with BW as a block. Each dietary treatment had eight replicates with four piglets per replicate. The piglets received a low-protein diet (LP, 18.27% CP), a high-protein diet (HP, 20.97% CP), and a high-protein diet supplemented with 300 mg/kg probiotics (HPPRO) for 21 days. The probiotics used in this study, provided by Kemin (Zhuhai, China) Technologies Co. Ltd., contained *B. subtilis PB6* spores at 2 × 10^11^ CFU/kg. All the protein-containing ingredients were analyzed for gross energy and crude proteins (Table 1). The LP diet was formulated to be similar to the typical CP level of commercial feed for weaned piglets. The CP level of the HP diet was calculated based on the average nitrogen requirements of growing pigs at 5–7 kg and 7–11 kg, as estimated by the National Research Council (11). The two diets were designed to be iso-energetic, and the CP levels of the diets were analyzed after feed preparation. All piglets were housed in an environmentally controlled room, and the temperature was maintained between 26 and 28°C. Piglets had free access to feed and water throughout the experimental period.

**Table 1:** Composition of ingredients and nutrient levels of the diet (as-fed basis).

Ingredients^a^, %	Low-protein diet	High-protein diet
Corn (7.32% CP)	26.67	21.92
Extruded corn (7.79% CP)	26.00	22.00
Dehulled soybean meal (44.14% CP)	6.10	15.30
Enzymatic soybean (38.5% CP)	5.00	5.00
Extruded soybean (34.99% CP)	5.00	5.00
Low-protein whey powder (2.00% CP)	10.00	10.00
Soybean concentrate protein (66.43% CP)	4.00	4.00
Fish meal (68.75% CP)	4.00	4.00
Whole milk powder (23.57% CP)	4.00	4.00
Soybean oil	0.85	1.00
Sucrose	4.00	4.00
_L_-Lysine·HCl (98%)	0.70	0.45
_DL_-Methionine (98.5%)	0.28	0.20
_L_-Threonine (98%)	0.29	0.17
_L_-Tryptophan (98%)	0.10	0.05
Choline chloride (50%)	0.16	0.16
Calcium carbonate	0.86	0.82
Dicalcium phosphate monohydrate	0.84	0.78
Sodium chloride	0.40	0.40
Acidifier^b^	0.50	0.50
Mineral premix^c^	0.20	0.20
Vitamin premix^d^	0.05	0.05
Total	100.00	100.00
Calculated nutrient levels		
Digestible energy, MCal/kg	3.58	3.58
Crude protein, %	18.01	21.00
Ca, %	0.83	0.83
Available P, %	0.43	0.43
SID Lys, %	1.43	1.43
SID Met, %	0.56	0.52
SID Met+Cys, %	0.78	0.78
SID Thr, %	0.84	0.84
SID Trp, %	0.27	0.27
Analyzed		
Gross energy, MCal/kg	3.98	4.00
Crude protein, %	18.27	20.97

a The crude protein values of corn, extruded corn, dehulled soybean meal, enzymatic soybean, extruded soybean, low-protein whey powder, soybean concentrate protein, fish meal, and whole milk powder were analyzed values.

b The acidifier (ACID LAC™) was provided by Kemin (China) Technologies Co., Ltd.

c Mineral premix provided per kilogram of feed: Fe, 100 mg; Cu, 6 mg; Mn, 4 mg; Zn, 100 mg; I, 0.14 mg; and Se, 0.3 mg.

d Vitamin premixes provided per kilogram of diet: vitamin A, 15,000 IU; vitamin D_3_, 5,000 IU; vitamin E, 40I U; vitamin K_3_, 5 mg; vitamin B_1_, 5 mg; vitamin B_2_, 12.5 mg; vitamin B_6_, 6 mg; vitamin B_12_, 0.06 mg; D-biotin, 0.25 mg; D-pantothenic acid, 25 mg; folic acid, 2.5 mg; and nicotinamide, 50 mg.

### 2.3 Growth performance and diarrhea scores

The BW and feed consumption of piglets were recorded weekly to calculate the average daily gain (ADG), average daily feed intake (ADFI), and the ratio of ADFI to ADG (F:G). Diarrhea scores were visually assessed 3 times a day as described in a previous study (12). Briefly, firm and well-formed feces were scored as 0; soft and formed feces were scored as 1; fluid and usually yellowish feces were scored as 2; and watery and projectile feces were scored as 3. The average diarrhea score = the sum of diarrhea scores/(number of piglets per pen × experimental days × assessed times per day).

### 2.4 Sample collection

The piglets were weighed individually on the morning of day 8 (6 a.m.) after an overnight fast. One pig per pen, closest to the pen mean, was then selected for blood sampling (*n* = 8). In total, 10 milliliters of blood samples from the jugular vein were collected into sodium heparinized tubes. The plasma samples were obtained by centrifuging blood samples at 3,000 *g* for 15 min and then stored at −20°C for later analysis.

To determine the apparent total tract digestibility (ATTD) of nutrients (gross energy, CP, DM, and amino acids), chromium oxide was added to the diets at 0.3% as an indigestible marker in the second week of the experiment. The first 4 days of this week were considered an adaptation period; therefore, fresh fecal samples were collected only during the last 3 days and pooled. The collected samples of feed and feces were stored at −20°C for later analysis.

After the 21-day dietary intervention, the piglets were weighed individually in the morning (6 a.m.) of day 22 after an overnight fast. Similarly, the piglet with BW closest to the average BW of this pen received an intramuscular injection of anesthetic (Shu Mianling II Injection, 0.1 mL/kg BW; Laboratory of Animal Disease, Chengdu, China) and was then euthanized. The ileal tissue samples of approximately 4 cm in length were opened longitudinally and were washed with physiological saline to remove chyme and stored immediately in liquid nitrogen. The colonic chyme was collected into sterile tubes and stored immediately in liquid nitrogen. All the samples stored in liquid nitrogen were then transferred to a − 80°C refrigerator for long-term storage.

### 2.5 Chemical analysis

Feed and fecal samples were dried at 65°C for 72 h, ground through a 0.42-mm sieve, and analyzed for DM according to AOAC (13) methods. CP was determined using the copper catalyst Kjeldahl method, and gross energy was determined using an automatic adiabatic oxygen bomb calorimeter (Parr 6,400, Parr Instrument Co., Moline, IL, United States). Amino acids, except tryptophan, were measured using an automatic amino acid analyzer (L-8900, Hitachi, Tokyo, Japan) after acidolysis for 24 h. Chromium was determined using a flame atomic absorption spectrophotometer (ContrAA 700, Analytik Jena, Jena, Germany). The ATTD was calculated according to the following equation: ATTD_nutrient_ = 1 − (Cr_diet_ × Nutrient_feces_)/(Cr_feces_ × Nutrient_diet_).

### 2.6 Plasma metabolites

The frozen plasma samples were thawed on the ice and centrifuged at 3000 *g* for 5 min. The supernatant was collected to determine the concentration of haptoglobin and pig major acute-phase protein (Pig-MAP) using enzyme-linked immunosorbent assay (ELISA), according to the kit instructions (Nanjing Jiancheng Bioengineering Institute, Nanjing, China).

### 2.7 RNA extraction and real-time quantitative PCR

The total RNA from the ileal tissue was extracted using a Trizol reagent (TaKaRa Biotechnology, Dalian, China). Then, the concentration and purity of the extracted RNA were measured using a nucleic acid analyzer (Beckman DU-800; Beckman Coulter, Inc., Brea, CA). Reverse transcription and real-time quantitative polymerase chain reaction (RT-qPCR) were performed according to the kit instructions (Vazyme, Nanjing, China). The total PCR reaction system was 20-μL, consisting of 0.4-μl forward primer, 0.4-μl reverse primer, 2.0-μl cDNA, 7.2-μl ddH_2_O, and 10.0-μl Master Mix (Nanjing, China). *β*-Actin was treated as a housekeeping gene to normalize the expression of target genes according to the 2^−ΔΔCt^ method. The PCR primers used in this study are listed in Supplementary Table S1.

### 2.8 Sequencing of gut microbiome

Genomic DNA was extracted from the colonic chyme samples using the Mo Bio Power DNA Isolation Kit (Mo BIO, San Diego, USA). Then, 1% agarose gels were used to monitor DNA concentration and purity. The v4 hypervariable regions of 16S rRNA were amplified using primers 515F (GTGCCAGCMGCCGCGGTAA) and 806R (GGACTACHVGGGTWTCTAAT), and the amplicon pyrosequencing was carried out on an Illumina HiSeq PE250 platform (Illumina) by Novogene (Novogene, Beijing, China). Sequencing libraries were generated using the Ion Plus Fragment Library Kit 48 rxn (Thermo Scientific, Massachusetts, USA). The Ribosomal Database Project Classifier (version 2.2) was used to assign taxonomic rank. Operational taxonomic units (OTUs) were clustered at 97% sequence identity (sequences with ≥97% similarity were assigned to the same OTU). The relative abundance of each OTU was examined at different taxonomic levels. Diversity within communities (*α*-diversity) calculations and taxonomic community assessments were performed using Mothur 1.30.2 and Qiime 1.9.1. Principal coordinates analysis (PCoA) plots were produced using the weighted UniFrac metrics. The linear discriminant analysis (LDA) effect size (LEfSe) method was performed to elucidate the difference between treatments with LDA scores above 2.5.

### 2.9 Quantification of short-chain fatty acids

The concentrations of short-chain fatty acids (SCFAs, such as acetate, propionate, and butyrate) in the colonic digesta were determined by gas chromatography (Varian CP-3800). Approximately 0.5 g of chyme matter was diluted with 1.5 mL of ultrapure water and centrifuged at 10,000 *g* for 15 min. The 1.0 mL of supernatant was mixed with 0.2 mL of 25% metaphosphoric acid solution and 23.3 μL of 210 mmol/L crotonic acid, and then placed at 4°C for 30 min before centrifuging at 10,000 *g* for 10 min. The 300 μL of supernatant was mixed with 900 μL of methanol (1:3 dilution), centrifuged at 10,000 *g* for 15 min, and filtered using a 0.22-μm filter (Millipore Co., Bedford, MA, United States) before being manually applied onto the gas chromatograph for quantification.

### 2.10 Statistical analysis

The homogeneity of variance and normality of the data were evaluated using the Shapiro–Wilk test and Levene’s test procedures of the SAS 9.4 (SAS Institute, Inc., Cary, NC, United States) software package. For growth performance, diarrhea scores, and ATTD, pens served as the experimental unit, and piglet data were reported as the mean of the pen. The data were analyzed using the PROC “MIXED” procedure, with dietary treatment as a fixed effect and initial BW as a random effect, using the following statistical model:


$$Y=\mu +\alpha_{i}+\upsilon_{j}+\varepsilon_{ij}$$

where *Y* is the parameter to be tested, *μ* is the mean, *α_i_* is the effect of the diet (*i* = 1, 2), *υ_j_* is the random effect of the BW, and *ε_ij_* is the error term. The differences in the abundance of microbial taxa among the three groups were performed using the Kruskal–Wallis test. The differential bacterial taxa among the groups were identified using linear discriminant analysis (LDA) by LEfSe. Pearson correlation analysis was applied to explore the potential association between SCFA concentration and immune-related gene expressions. Spearman correlation was applied to the microbiota data and individual SCFA data. For all statistical analyses, the statistical significance was declared at *p* < 0.05 and trends at 0.05 ≤ *p* < 0.10.

## 3 Results

### 3.1 Growth performance

As shown in Table 2, piglets fed the HPPRO diet had elevated ADG and ADFI during the second and third weeks of the experiment and throughout the experimental period (*p* < 0.05), compared to piglets fed the LP diet. Similarly, piglets fed the HPPRO diet had increased BW on day 15 of the experiment (+7.4%) and at the end of it (+12.2%) than those fed the LP diet (*p* < 0.01). In parallel, piglets fed the HPPRO diet had a lower F:G during the second week (*p* < 0.05) and tended to have a lower F:G throughout the experimental period (*p* = 0.05) than the LP piglets. The growth performance of piglets fed the HP diet showed no significant differences throughout the experiment from that of piglets fed the LP and HPPRO diets (*p* > 0.05).

**Table 2:** Growth performance in weaned piglets.

	LP	HP	HPPRO	SEM	*p*-value
BW, kg					
Day 1	6.13	6.12	6.12	0.17	0.81
Day 8	6.44	6.45	6.48	0.16	0.79
Day 15	7.18^b^	7.44^ab^	7.71^a^	0.20	<0.01
Day 22	8.64^b^	9.22^ab^	9.69^a^	0.27	<0.01
ADG, g/d					
Days 1–7	44	46	52	4.42	0.69
Days 8–14	106^b^	141^ab^	176^a^	10.07	<0.01
Days 15–21	208^b^	255^ab^	283^a^	13.40	0.04
Days 1–21	119^b^	148^ab^	170^a^	7.38	0.01
ADFI, g/day					
Days 1–7	139	130	141	5.19	0.62
Days 8–14	264^b^	315^ab^	348^a^	14.72	0.01
Days 15–21	397^b^	459^ab^	509^a^	18.28	<0.01
Days 1–21	266^b^	301^ab^	333^a^	11.63	0.01
F: G					
Days 1–7	2.94	2.84	2.76	0.20	0.90
Days 8–14	2.59^a^	2.38^ab^	2.00^b^	0.10	0.02
Days 15–21	1.92	1.83	1.86	0.05	0.74
Days 1–21	2.24	2.07	1.98	0.05	0.05

LP, low-protein diet; HP, high-protein diet; HPPRO, high-protein diet with probiotics; ADG, average daily gain; ADFI, average daily feed intake; F:G, the ratio of ADFI to ADG (feed to gain ratio). Values within a row with different superscripts differ significantly at *p* < 0.05. *n* = 8.

### 3.2 Diarrhea scores

**Figure 1:**
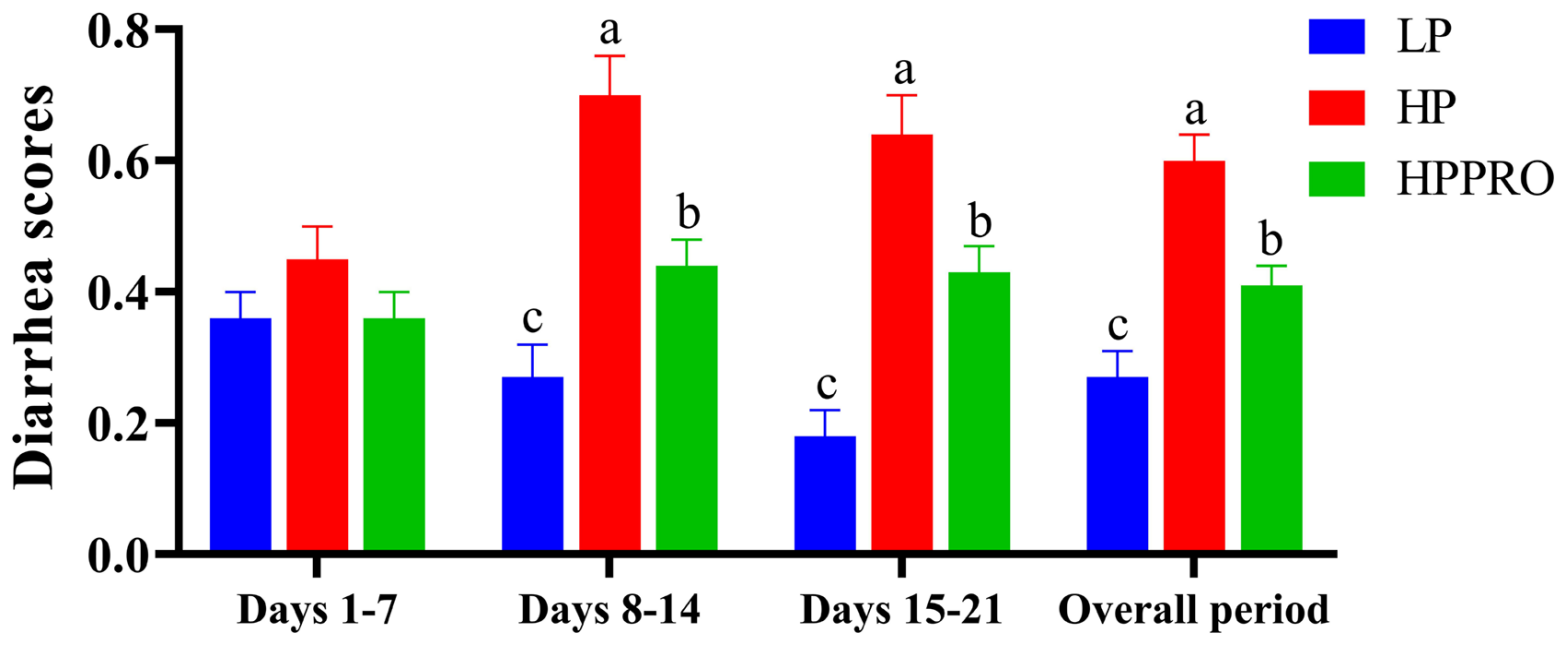
Diarrhea scores in weaned piglets. LP, low-protein diet; HP, high-protein diet; HPPRO, high-protein diet with probiotics. ^a,b,c^ Values without a common letter differ significantly (*p* < 0.05). *n* = 8. Diarrhea score: 0, normal; 1, soft feces; 2, mild diarrhea; and 3, severe diarrhea.

The results of the diarrhea scores are shown in Figure 1. The HP and HPPRO diets markedly increased the diarrhea scores of the piglets during the second and third weeks of the experiment and throughout the experimental period, as compared to the LP diet (*p* < 0.01). In addition, the piglets fed the HPPRO diet had lower diarrhea scores than those fed the HP diet during the second and third weeks of the experiment and throughout the experimental period (*p* < 0.01).

### 3.3 ATTD of nutrients and amino acids

As shown in Table 3, the piglets fed the LP and HPPRO diets had elevated ATTD of gross energy, DM, and total amino acids compared to the piglets fed the HP diet (*p* < 0.01). Meanwhile, the ATTD of CP in piglets fed the HPPRO diet was higher than in piglets fed the LP and HPPRO diets (*p* < 0.01). Specifically, the ATTD of Lys, Met, Thr, Ile, Val, and Ala in the LP and HPPRO groups was higher than that of the HP group (*p* < 0.01). The ATTD of Phe, Asp., Glu, Pro, Ser, and Tyr in the HPPRO group was higher than that of the LP and HP groups (*p* < 0.01). The ATTD of Cys in the LP group was higher than that of the HP group (*p* < 0.05). The ATTD of Arg in the LP and HPPRO groups tended to be higher than that of the HP group (*p* = 0.09).

**Table 3:** Apparent total tract digestibility of nutrients and amino acids in weaned piglets.

	LP	HP	HPPRO	SEM	*p*-value
Gross energy	83.17^a^	80.86^b^	85.07^a^	0.51	<0.01
Dry matter	83.89^a^	81.73^b^	85.29^a^	0.44	<0.01
Crude protein	72.81^b^	71.37^b^	76.97^a^	0.80	<0.01
Total amino acids	77.61^a^	74.53^b^	79.96^a^	0.70	<0.01
Essential amino acids					
Lys	84.28^a^	78.23^b^	83.51^a^	0.71	<0.01
Met	81.94^a^	74.81^b^	79.27^a^	0.82	<0.01
Thr	76.76^a^	70.65^b^	76.93^a^	0.83	<0.01
Leu	73.37^ab^	70.43^b^	76.36^a^	0.81	<0.01
Ile	69.57^a^	65.82^b^	72.80^a^	0.95	<0.01
Val	69.92^a^	65.90^b^	72.83^a^	0.94	<0.01
His	85.25^ab^	84.17^b^	87.36^a^	0.48	0.01
Phe	72.45^b^	69.96^b^	76.07^a^	0.82	<0.01
Non-essential amino acids					
Ala	69.12^a^	63.80^b^	71.16^a^	1.00	<0.01
Asp	74.87^b^	72.39^b^	78.74^a^	0.79	<0.01
Arg	87.68	87.17	89.31	0.42	0.09
Cys	82.58^a^	76.88^b^	81.64^ab^	1.04	0.04
Glu	82.78^b^	81.03^b^	85.52^a^	0.57	<0.01
Gly	71.23^ab^	68.02^b^	74.55^a^	0.85	<0.01
Pro	81.26^b^	79.75^b^	83.73^a^	0.59	<0.01
Ser	75.98^b^	74.00^b^	79.87^a^	0.75	<0.01
Tyr	68.59^b^	68.24^b^	74.58^a^	1.00	<0.01

LP, low-protein diet; HP, high-protein diet; HPPRO, high-protein diet with probiotics. Values within a row with different superscripts differ significantly at *p* < 0.05. *n* = 8.

### 3.4 Stress-related indicators

**Figure 2:**
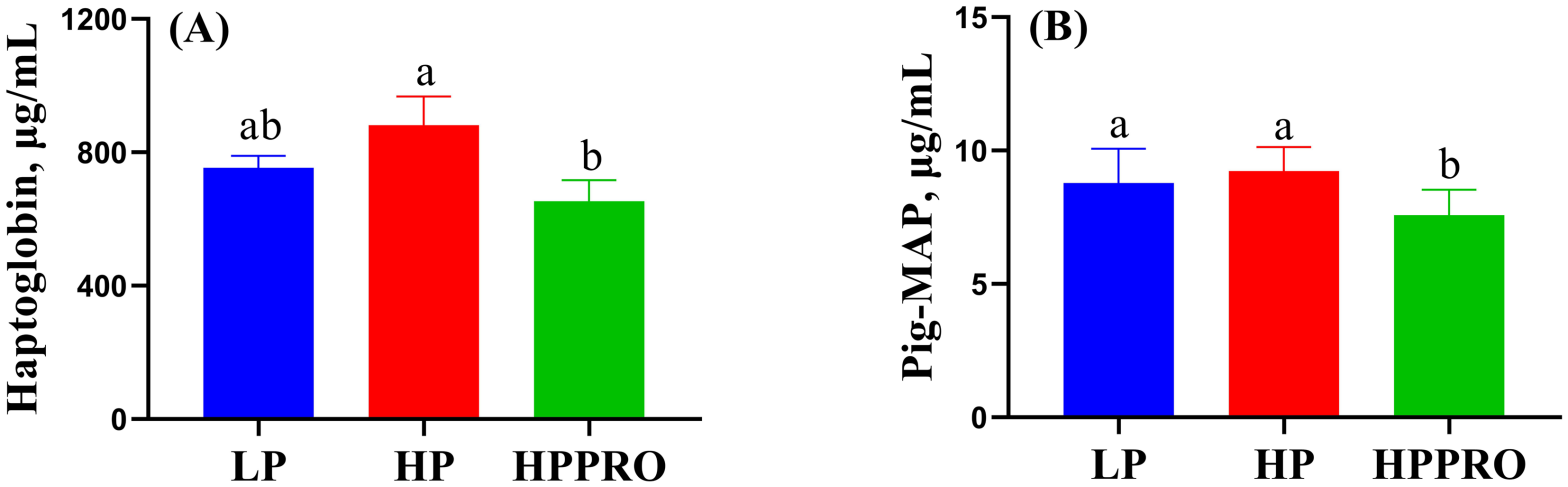
The stress-related indicators in weaned piglets. LP, low-protein diet; HP, high-protein diet; HPPRO, high-protein diet with probiotics; pig major acute-phase protein. ^a,b^ Values without a common letter differ significantly (*p* < 0.05). *n* = 8.

Piglets from the HPPRO group had lower plasma concentrations of haptoglobin than HP piglets (Figure 2A; *p* < 0.05). Meanwhile, piglets fed the HPPRO diet had decreased plasma concentrations of Pig-MAP compared to those fed the LP and HP diets (Figure 2B; *p* < 0.01).

### 3.5 Gene expression

**Figure 3:**
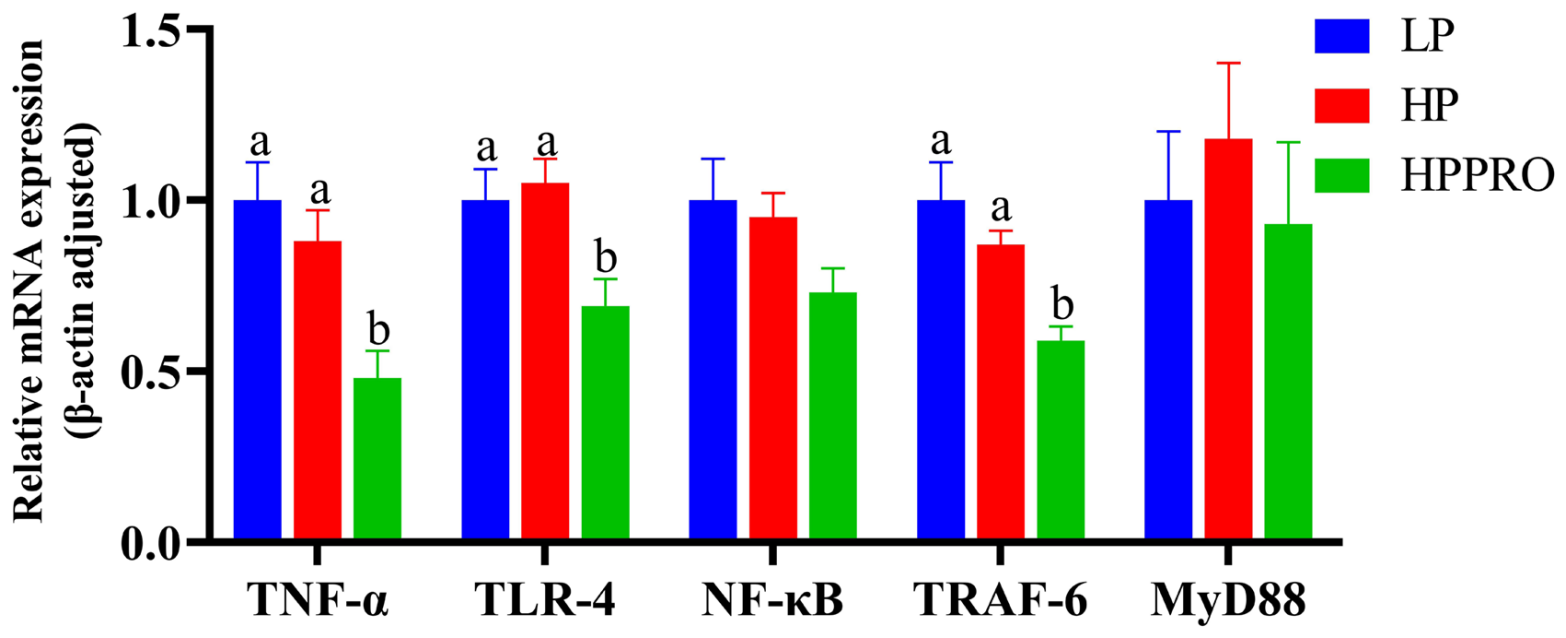
Gene expression in the ileum of weaned piglets. LP, low-protein diet; HP, high-protein diet; HPPRO, high-protein diet with probiotics; *TNF-α*, tumor necrosis factor-α; *TLR-4*, toll-like receptor-4; *NF-κB*, nuclear factor kappa B; *TRAF-6*, TNF receptor-associated factor-6; *MyD88*, Myeloid differentiation-88. ^a,b,c^ Values without a common letter differ significantly (*p* < 0.05). *n* = 8.

As shown in Figure 3, compared to those fed the LP and HP diets, piglets fed the HPPRO diet had lower mRNA expressions of *TNF-α*, *TLR-4*, and *TRAF-6* in the ileum (*p* < 0.01).

### 3.6 Gut microbiota of colonic digesta

**Figure 4:**
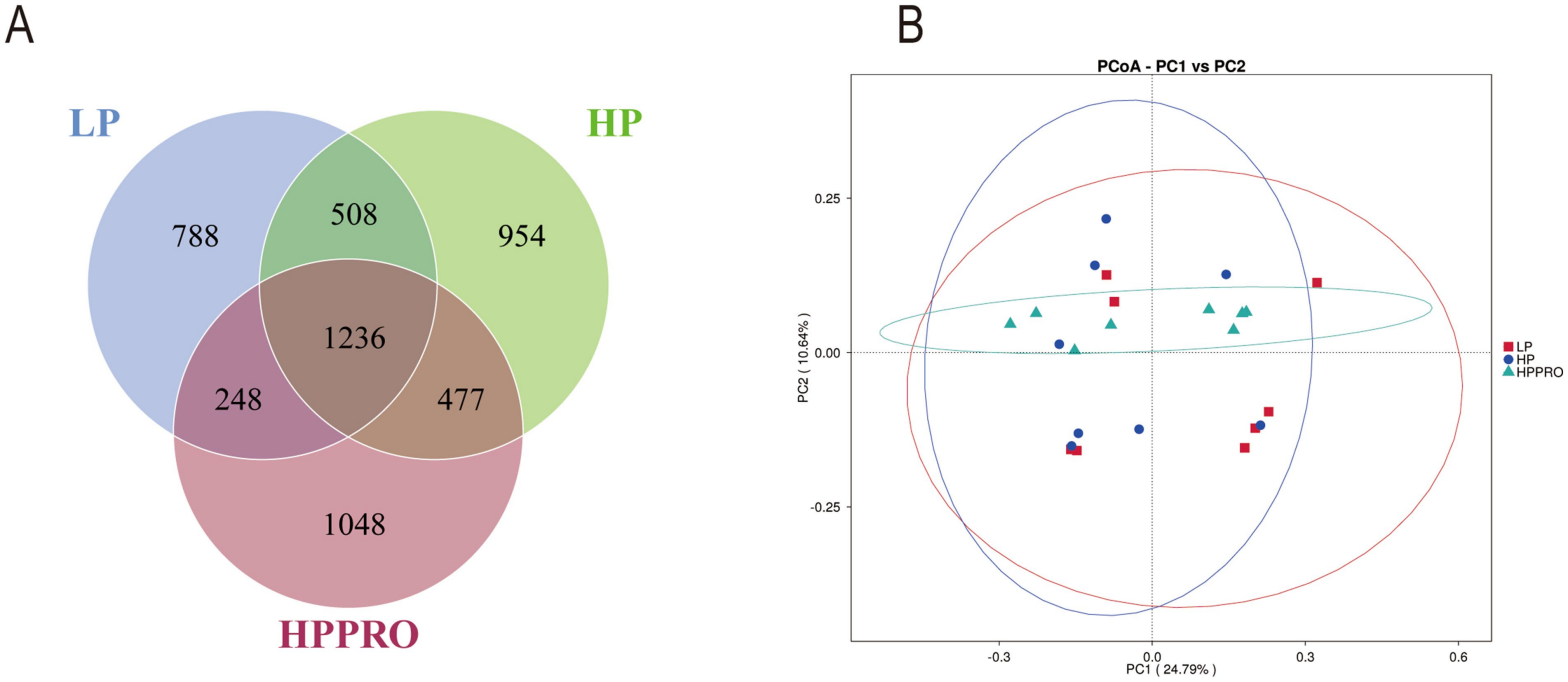
Gut microbiota of the colonic chyme in weaned piglets fed different diets. **(A)** Venn diagram based on OTUs; **(B)** *β*-diversity using PCoA. *n* = 8.

Across all 24 colonic chyme samples, a total of 1,933,314 high-quality sequences were classified as being bacteria, with an average of 80,554 ± 906 sequences per sample. A total of 5,259 OTUs were identified by a nucleotide sequence identity of 97% between reads. A Venn diagram was used to demonstrate the results of shared richness between samples. As shown in Figure 4A, 1236 OTUs were shared in three groups, while 788 were unique in the LP group, 954 were unique in the HP group and 1,048 were unique in the HPPRO group. To demonstrate the separation of bacterial community composition in two groups, unweighted-UniFrac dissimilarities were calculated using the first two principal component scores of PC1 and PC2 (24.79 and 10.64%, respectively) of the explained variance and finally displayed by PCoA (Figure 4B).

**Figure 5:**
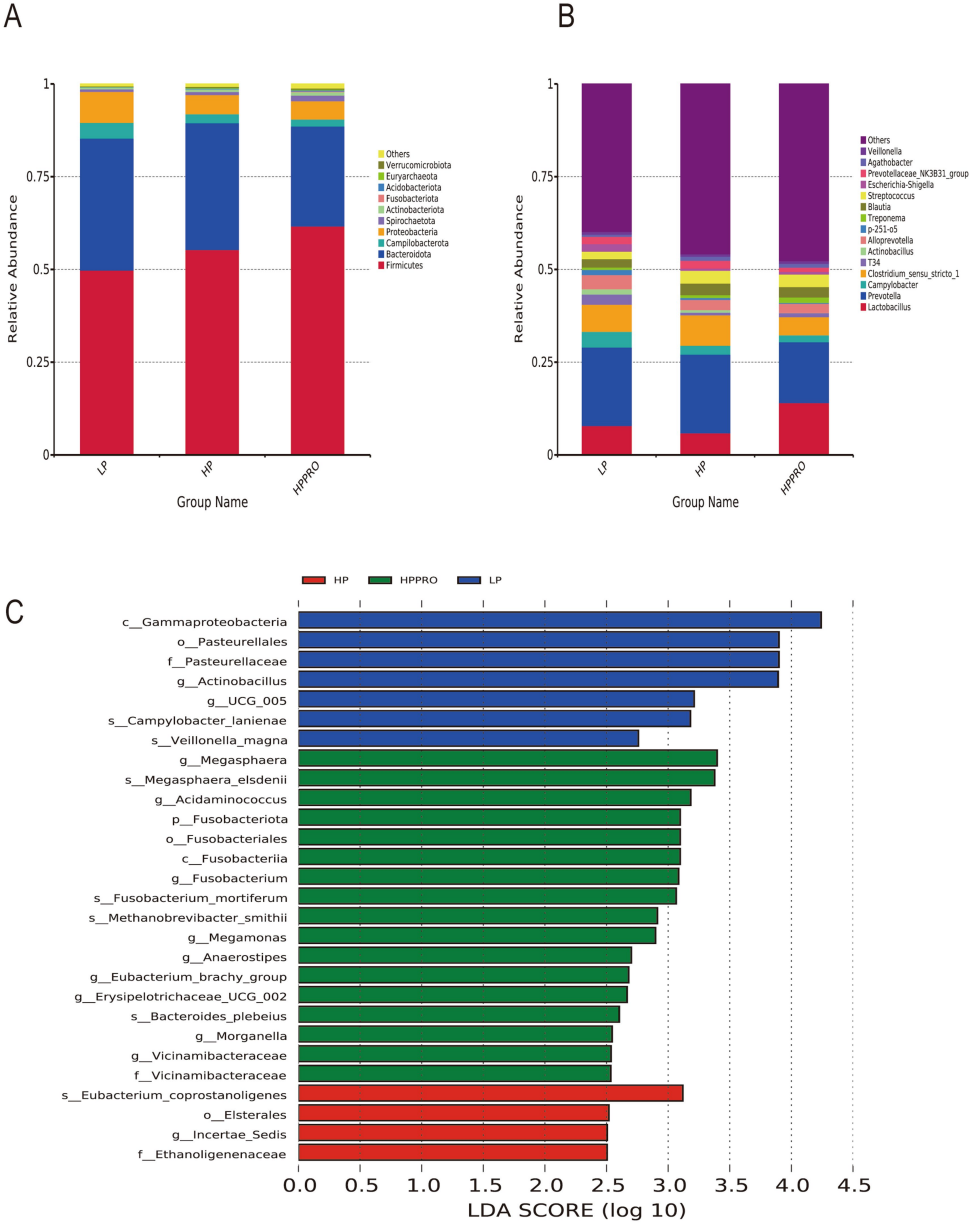
Gut microbiota of the colonic chyme in weaned piglets. Relative abundance at the phylum level **(A)** and genus level **(B)**; **(C)** Differentially abundant taxa in weaned piglets based on LEfSe analysis (LDA score > 2.5). LP, low-protein diet; HP, high-protein diet; HPPRO, high-protein diet with probiotics. *n* = 8.

No significant difference was found among the groups in *α*-diversity (Supplementary Table S2). The microbiota data cover 39 phyla and were further divided into 718 genera. At the phylum level, *Firmicutes* and *Bacteroidetes* were the two predominant bacterial taxa in the gut microbiota (Figure 5A). HPPRO piglets had a higher relative abundance of *Firmicutes*, but a lower relative abundance of *Bacteroidota* than LP piglets (*p* < 0.05). The top 15 genera are shown in Figure 5B. The LEfSe approach was conducted to discover high-dimensional biological markers among the groups. Seven taxa exhibited higher abundance in the LP group, while 4 taxa and 17 taxa were enriched in the HP and HPPRO groups, respectively (Figure 5C).

### 3.7 Concentrations of SCFA in colonic digesta

**Figure 6:**
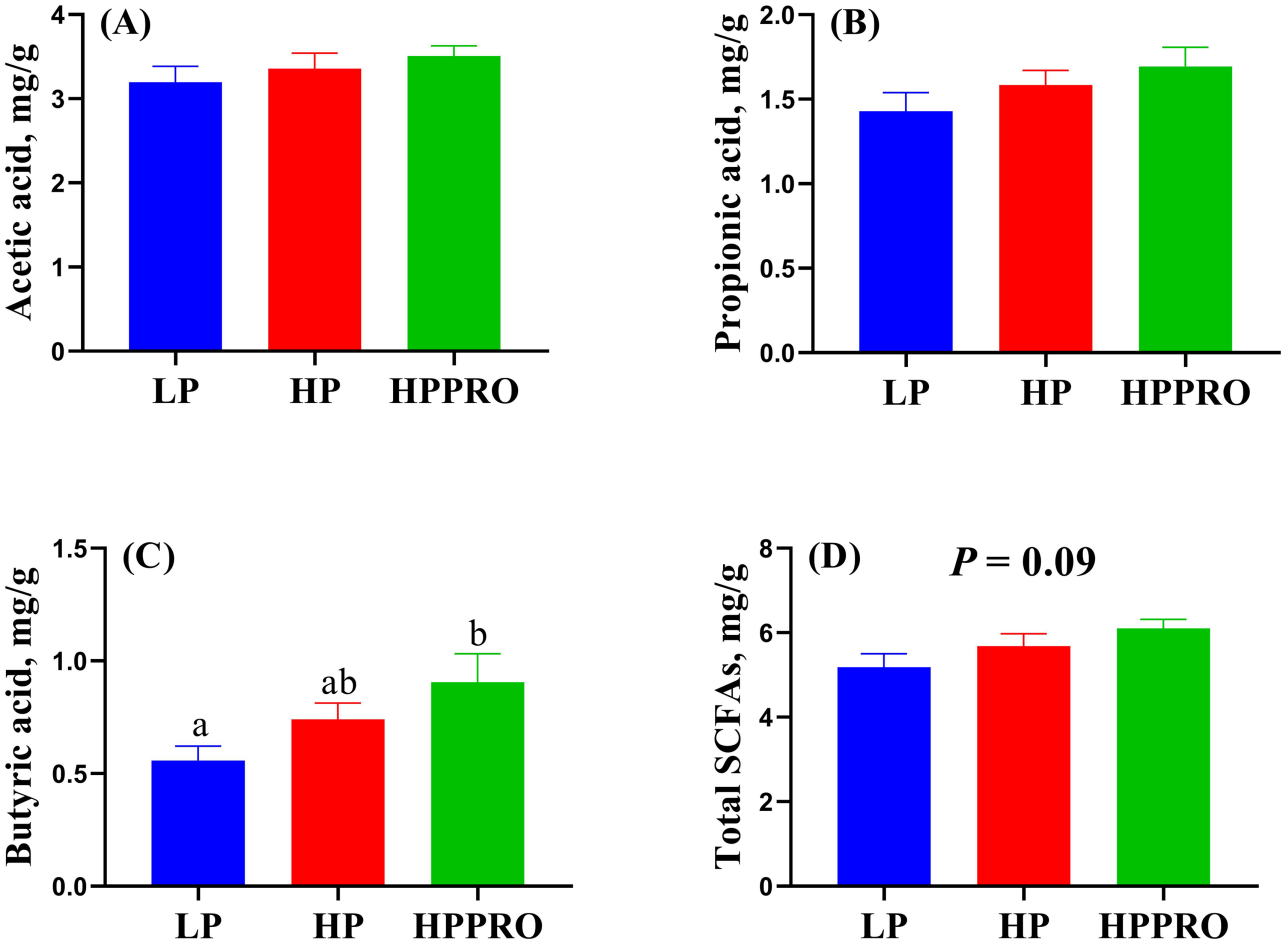
Short-chain fatty acids in the colonic digesta of weaned piglets. Total SCFAs are the sum of acetic acid, propionic acid, and butyric acid. LP, low-protein diet; HP, high-protein diet; HPPRO, high-protein diet with probiotics. *n* = 8. ^a,b^ Values without a common letter differ significantly (*P* < 0.05).

The piglets fed the HPPRO diet had increased levels of butyric acid (Figure 6C, *p* < 0.05) and tended to have higher levels of total SCFA (Figure 6D, *p* = 0.09) in the colonic digesta compared to the piglets fed with the LP diet. The levels of acetic acid and propionic acid in the colonic digesta did not markedly differ among groups (Figures 6A,B, *p* > 0.05).

### 3.8 Correlations

**Figure 7:**
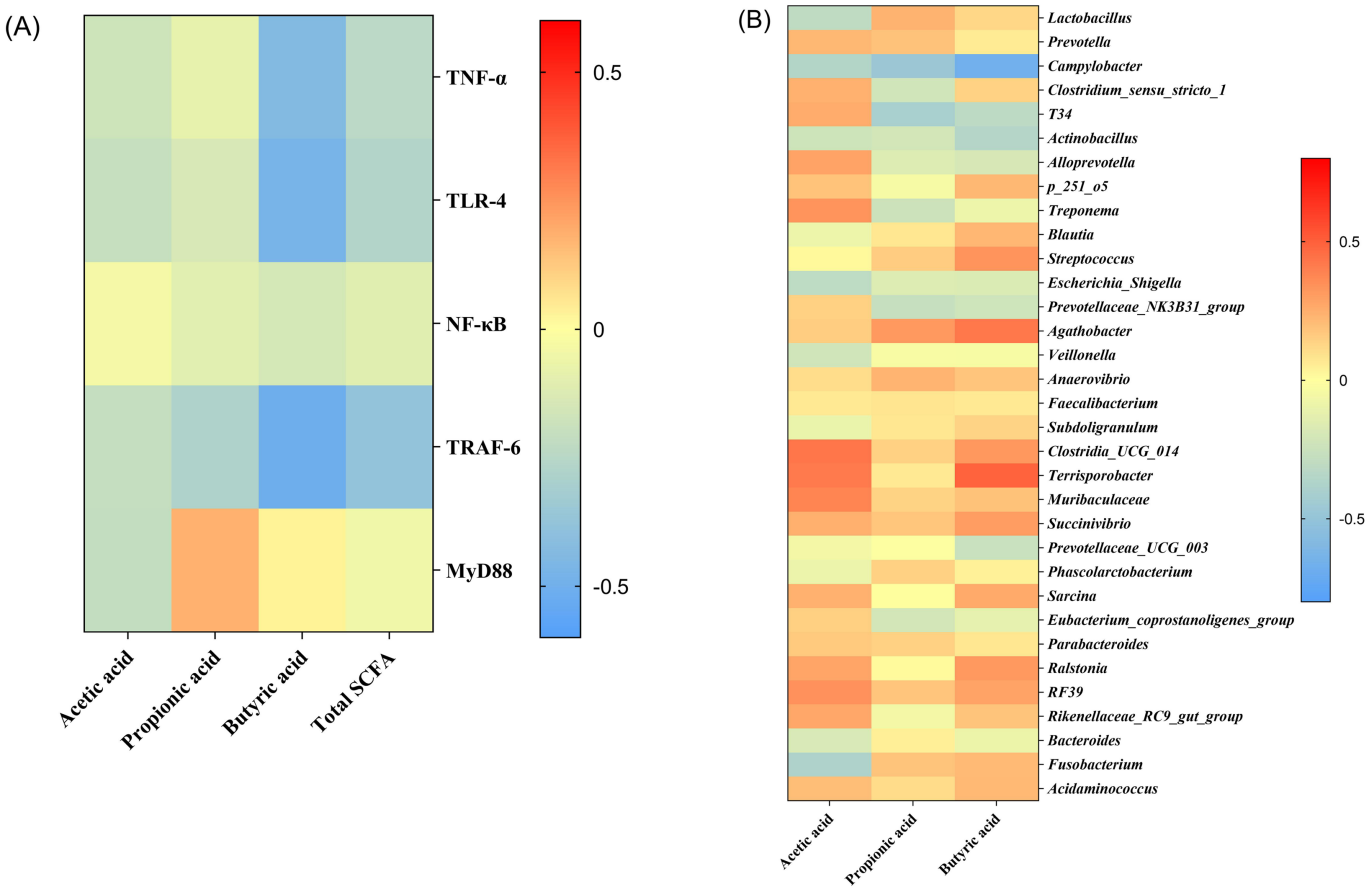
Heatmaps of the correlation analysis. Associations between short-chain fatty acids and immune-related gene expressions based on Pearson correlation analysis **(A)**; Associations between gut microbiota and short-chain fatty acids based on Spearman’s correlation analysis **(B)**. Correlation coefficients were colored according to the scale listed on the right.

Pearson correlation analysis was used to find the association between SCFA concentration and immune-related gene expressions (Figure 7A). The butyric acid was negatively correlated with the mRNA expressions of TNF-*α*, TLR-4, and TRAF-6.

Correlations between the abundance of specific taxa and SCFA using Spearman correlation analysis were shown as heatmaps (Figure 7B). Multiple taxa were correlated with SCFA in abundance. Specifically, *Campylobacter* was negatively correlated with concentrations of acetic acid, propionic acid, and butyric acid, while *Terrisporobacter* was positively correlated with concentrations of acetic acid and butyric acid. *Clostridia_UCG_014* and *Muribaculaceae* showed a positive correlation with acetic acid concentration. In addition, *T34* was negatively correlated with propionic acid concentration. *Actinobacillus* was negatively correlated with butyric acid concentration, while *Agathobacter* was positively correlated with butyric acid concentration.

## 4 Discussion

Dietary protein and its metabolites, small peptides, and amino acids play an important role in cell proliferation and tissue synthesis. Insufficient protein intake compromises the growth performance of weaned piglets. Low-protein diets supplemented with synthesized amino acids have been widely used to reduce postweaning diarrhea in piglets and the use of protein ingredients in feed. Although the amino acid pattern has been considered and crystalline amino acids are added to satisfy the requirement of amino acids, the reduced intact proteins (protein-bound amino acids) and small peptides are inevitable in a low-protein diet due to the reduced dietary protein. Our previous study indicated that intact protein is superior to free amino acids for whole-body protein synthesis (14). Compared to free amino acids, the intact protein or protein-bound amino acids elevate the nitrogen retention rate and protein homeostasis, which is been associated with amino acid availability in the form of di- and tripeptides digested from intact protein (14). Although no statistically significant difference was observed between LP and HP piglets in growth performance, the HP piglets had numerical increases in ADG (+24%) and ADFI (+13%). In the present study, the energy and essential amino acid levels between the low-protein and high-protein diets were comparable, implying that the better growth performance of piglets in the HP and HPPRO groups may be attributed to the higher content of intact protein, which is required for maintaining the optimal growth and immune function in pigs (4).

Despite this, it should be stressed that the digestibility of gross energy, DM, CP, and amino acids in the HP group was lower than that in the LP group, indicating that more undigested protein is generated due to the higher dietary protein content. Nevertheless, the decreased nutrient digestibility of the HP diet was reversed by *B. subtilis PB6* supplementation. Consistent with this, a previous study reported that dietary *B. subtilis* DSM32315 increased the digestibility of CP, DM, and gross energy in weaned piglets (15). Supplementation of *B. subtilis* in the diet could reduce the pH value of jejunal and ileal digesta (15), creating an acidic environment conducive to protein degradation and absorption. Meanwhile, the protease could enhance the deposition of both protein and energy by hydrolyzing the less digestible protein (16), which may explain the higher energy digestibility in HPPRO piglets. In addition, we have previously found that dietary *B. subtilis PB6* supplementation increased the activities of disaccharidases (maltase and sucrase) in the jejunum of piglets (17), indicating better digestive capability in response to *B. subtilis PB6* supplementation. These results indicate that *B. subtilis PB6* improves nutrient availability in weaned piglets.

In this study, we observed that the piglets on the HP and HPPRO diets had more severe diarrhea compared to the LP piglets, implying an impaired intestinal mucosal barrier and increased intestinal permeability due to higher protein intake. However, it should be noted that HPPRO piglets had markedly lower diarrhea scores than HP piglets, further suggesting the beneficial role of *B. subtilis PB6* in maintaining intestinal homeostasis in piglets fed a high-protein diet. The tight junction proteins, such as zonula occludens-1 (ZO-1), occludin, and claudin-1, modulate intestinal barrier function. Our previous study reported that *B. subtilis* BP6 increased the relative protein expression of ZO-1 and claudin-1 in the ileum of pigs (17). Similarly, *B. subtilis* treatment reduced plasma diamine oxidase activity (10), a sensitive indicator of intestinal permeability. Therefore, *B. subtilis PB6* could potentially improve the integrity of the intestinal barrier, which may contribute to alleviating diarrhea.

The profound link between diet and gut microbiota has been widely reported. It is accepted that diet plays a vital role in shaping the microbial communities of the host (18). Conversely, the gut microbiota is also involved in the retention of nutrients (19). In this study, we performed 16S rRNA sequencing to investigate the gut microbiota responses to dietary treatments. The diversity and richness of the gut microbiota did not markedly differ among the groups. A previous study reported that the ratio of *Firmicutes* to *Bacteroidetes* was positively correlated with an increased body weight gain (20). In the current study, consistently, the increased abundance of *Firmicutes*, decreased abundance of *Bacteroidetes*, and the highest *Firmicutes*/*Bacteroidetes* ratio among the three groups explained the highest BW and ADG in HPPRO piglets. It is reported that gut microbiota mediate dietary protein metabolism and nitrogen recycling in the intestine (21). To elucidate the differences in gut microbiota composition between treatments, the LEfSe method was conducted to analyze the enriched bacteria in each group. The genera of *Bacteroides*, *Fusobacterium*, *Clostridium*, and *Lactobacillus* possess proteolytic activity in the hindgut (21, 22). In addition, the g_*Acidaminococcus* and s_*Veillonella magna*, which are enriched in the colonic chyme of HPPRO and LP piglets respectively, are classified as *Clostridium* (23). In this study, the increased abundances of s_*Bacteroides*_*plebeius*, g_*Fusobacterium*, and g_*Acidaminococcus* in the HPPRO group may favor the digestion and absorption of protein, which was consistent with the highest CP digestibility in HPPRO piglets. In contrast, HP piglets had an enriched abundance of s_*Eubacterium*_*coprostanoligenes*, which ferment sulfur-containing amino acids to generate hydrogen sulfide and impair the gut barrier (24). Collectively, the altered gut microbiota may influence protein retention and ultimately impact piglet growth.

Acute-phase proteins (APPs), the proteins that respond to inflammation caused by stress or infection, are commonly used as markers of inflammatory problems or disease (25). In pigs, haptoglobin and Pig-MAP are the two significant APPs that are increased in experimental acute-phase models (25). In this study, HPPRO piglets had the lowest haptoglobin and Pig-MAP levels among the groups, indicating that the comprehensive stress induced by weaning and diet was alleviated by *B. subtilis PB6* supplementation. The activation of the TLR-4-mediated nuclear factor kappaB (NF-κB)–TNF-*α* signaling pathway in immune cells stimulates APP production (26, 27). The recruitment of MyD88 to intracellular fragments activates TLR-4 (26), which, via TRAF-6, an essential adapter protein that mediates the transduction of TLR-4/Myd88 signals, stimulates the NF-κB signaling cascade and translocases in the nucleus (28), and thereby modulates the expression of TNF-*α*. In this study, the decreased mRNA expressions of TLR-4, TRAF-6, and TNF-*α* in the HPPRO group further indicated that dietary supplementation with *B. subtilis PB6* ameliorated potential inflammatory lesions in weaned piglets. Short-chain fatty acids (SCFAs) have been widely reported to regulate host immunity and intestinal health (29). In this study, HPPRO piglets had higher butyric acid levels than LP piglets. The genera g_*Anaerostipes* and g_*Megasphaera*, which produce SCFAs, especially butyric acids (20, 30), showed increased abundance in HPPRO piglets. Notably, a previous study demonstrated that butyrate inhibits intestinal inflammatory responses and reduces proinflammatory factor production by inhibiting innate immune responses and inhibiting macrophage TNF-α (31). In support, our correlation analysis showed that the concentration of butyric acid was negatively correlated with the mRNA expressions of TLR-4, TRAF-6, and TNF-α, further confirmed the beneficial effect of butyric acid on the regulation of inflammation.

## 5 Conclusion

In this study, piglets fed the HP diet had higher diarrhea scores, which may be associated with decreased nutrient digestibility and altered gut microbiota composition; however, the inclusion of 300 mg/kg *B. subtilis PB6* in a high-protein diet improved nutrient digestibility, ameliorated diarrhea, and stress-related indicators, resulting in better growth performance and intestinal health.

## Data Availability

The data presented in the study are deposited in the NCBI repository, accession number PRJNA1215014. The original contributions presented in the study are included in the article/supplementary material, further inquiries can be directed to the corresponding author/s.
