# Supplementary material for: Dietary supplementation with Bacillus subtilis PB6 alleviates diarrhea and improves growth performance and immune function in weaned piglets fed a high-protein diet
Source: Front Vet Sci. 2025 Feb 18;12:1525354. doi: 10.3389/fvets.2025.1525354 (PMC11880896; doi:10.3389/fvets.2025.1525354)
Supplement: Supplementary file 1 [file Table_1.DOCX]

**Supplementary material**

Supplementary Table S1. Primer sequences of target and reference genes

| **Gene^1^** | **Primer sequence (5'→3')** | **Accession NO.** |
| --- | --- | --- |
| β-actin | F:GGCGCCCAGCACGAT  R:CCGATCCACACGGAGTACTTG | DQ845171.1 |
| TLR-4 | F:TTACAGAAGCTGGTTGCCGT  R:TCCAGGTTGGGCAGGTTAGA | GQ304754 |
| MyD88 | F:GTGCCGTCGGATGGTAGTG  R:TCTGGAAGTCACATTCCTTGCTT | NM001099923 |
| TRAF-6 | F:GCTGCATCTATGGCATTTGAAG  R:CCACAGATAACATTTGCCAAAGG | AJ606305.1 |
| NF-κB | F:TGCTGGACCCAAGGACATG  R:CTCCCTTCTGCAACAACACGTA | AK348766.1 |
| TNF-α | F:CCACGTTGTAGCCAATGTCA  R:CAGCAAAGTCCAGATAGTCG | X57321 |

*TLR-4*, toll-like receptor-4; *TNF-α*, tumor necrosis factor-α; *NF-κB*, nuclear factor kappa B; *TRAF-6*, TNF receptor-associated factor-6; *MyD88*, Myeloid differentiation-88.

Supplementary Table S2. Alpha diversity of piglets fed different diets

|  | LP | HP | HPPRO | SEM | *P*-value |
| --- | --- | --- | --- | --- | --- |
| chao1 | 836.09 | 976.27 | 929.79 | 52.94 | 0.57 |
| dominance | 0.04 | 0.03 | 0.04 | 0.005 | 0.50 |
| observed_otus | 828.13 | 971.38 | 925.12 | 53.46 | 0.56 |
| pielou_e | 0.71 | 0.73 | 0.73 | 0.01 | 0.61 |
| shannon | 6.80 | 7.26 | 7.13 | 0.17 | 0.54 |
| simpson | 0.96 | 0.97 | 0.97 | 0.005 | 0.13 |

LP, low-protein diet; HP, high-protein diet; HPPRO, high-protein diet with probiotics.
